# Phenotypic and genomic analysis of the hypervirulent ST22 methicillin-resistant *Staphylococcus aureus* in China

**DOI:** 10.1128/msystems.01242-22

**Published:** 2023-05-15

**Authors:** Huilin Zhao, Xiaocui Wu, Bingjie Wang, Li Shen, Lulin Rao, Xinyi Wang, Jiao Zhang, Yanghua Xiao, Yanlei Xu, Jingyi Yu, Yinjuan Guo, Ying Zhou, Baoshan Wan, Chunyang Wu, Liang Chen, Fangyou Yu

**Affiliations:** 1 Department of Clinical Laboratory, Shanghai Pulmonary Hospital, School of Medicine, Tongji University, Shanghai, China; 2 First Affiliated Hospital of Wenzhou Medical University, Wenzhou, China; 3 Hackensack Meridian Health Center for Discovery and Innovation, Nutley, New Jersey, USA; 4 Hackensack Meridian School of Medicine, Nutley, New Jersey, USA; Zhujiang Hospital, Southern Medical University, Guangzhou, Guangdong, China

**Keywords:** ST22, methicillin-resistant *Staphylococcus aureus*, genomic evolution, SCC*mec*IVa-t309, virulence, *pvl*, *tst*

## Abstract

ST22 MRSA (methicillin-resistant *Staphylococcus aureus*) strains are only sporadically reported in China. Through the phylogenetic reconstruction of 30 ST22 strains from China and 480 ST22 strains from global sources, we found that the global ST22 strains can be divided into three clades (I, II, and III). The China ST22 strains were found primarily in clade II (IIb and IIc) and also in clade III, indicating that the China ST22-MRSA clones have different origins. The China subclade IIb strains (SCC*mec* Vb-t309) may evolve from the native ST22 MSSA clone, while the China IIc strains may have spread from other countries. Subclade IIc (SCC*mec*IVa-t309) strains exhibited particularly strong lethality and invasiveness in *Galleria mellonella* infection and mouse skin abscess models in comparison to USA300 and other dominant China HA-MRSA (ST5 and ST239) or CA-MRSA (ST59) strains. This study described the emergence of a highly virulent ST22 MRSA subclade and improved our insight into the molecular epidemiology of ST22 strains in China.

**IMPORTANCE:**

ST22 is a successful hospital-associated MRSA lineage which first appeared in the United Kingdom as EMRSA-15. At present, ST22 MRSA clones are spreading rapidly around the world and even replaced other dominant clones in some regions. We placed the Chinese ST22 in the worldwide phylogeny of ST22, demonstrating a distinctive molecular epidemiology and to our knowledge, this is the first time that a novel clade of ST22 has been found in China. Among the 15 ST22 MRSA strains belonging to the novel clade, 14 ST22 SCC*mec*IVa strains from different regions carried both *pvl* and *tst* and displayed significantly higher *in vitro* and *in vivo* virulence in comparison to other clade/subclade ST22 strains as well as other common China HA-MRSA or CA-MRSA strains. The further spread of this subclade of strains could pose a serious threat to the health system in China and other regions.

Methicillin-resistant *Staphylococcus aureus* (MRSA) is a clinically common Gram-positive pathogen. About 0.9%–1.5% of healthy people are continuously colonized with MRSA, mainly in the anterior nostril, inguinal region, oropharynx, perineum, and axilla (1). The pathogenicity of MRSA is mainly attributed to the production of a variety of virulence factors, such as extracellular toxins, extracellular enzymes, and surface protein adhesion factors, that can cause various community-acquired infections and hospital-acquired infections, from relatively mild skin and soft tissue infections (SSTIs) to life-threatening pneumonia, osteoarthritis, endocarditis, and other severe infections (1, 2). Since the first detection in the United Kingdom in 1961, the number of MRSA infections has increased rapidly around the world.

The clonal structure of global epidemic MRSA populations is unstable, varying geographically and temporally. Among the MRSA clones detected globally, CC1, CC5, CC8, CC22, CC30, and CC45 are the most frequently reported. Among them, CC5 (e.g., ST5) and CC8 (e.g., ST8 and ST239) are the most common. The CC8-ST239 subgroup, CC5 (ST5) and CC22 (ST22) are the most frequently reported CC clone groups in some Asian countries (3 - 5).

ST22 is a successful hospital-associated MRSA lineage which first appeared in the United Kingdom as EMRSA-15. It can colonize and spread among hospital environments, humans, and companion pets. EMRSA-15 was the most rapidly transmitted hospital-acquired MRSA clone in Europe (6 - 8). At present, ST22 MRSA clone has spread from the United Kingdom to Europe, Asia, Africa, Australia/New Zealand, and the Middle East (9 - 12). Of particular concern is that ST22-MSRA, which contains the *lukS/F* gene (encoding the Panton–Valentine leucocidin), is causing an increased frequency of severe infections (13, 14). Furthermore, ST22-MRSA has a strong potential to replace other previously epidemic MRSA clones (15, 16). For instance, ST22-MRSA has a tendency to replace ST239-MRSA-SCC*mec*III in some countries such as Singapore, Indonesia, India, and Palestine (1, 17, 18).

The main prevalent clones of MRSA are ST5, ST59, and ST239 in China, but the frequency of these dominant clones has also undergone significant changes in recent years (18, 19). Although ST22 clones are frequently isolated in many countries, ST22 MRSA was rarely detected in China until recent years. It is worth noting that the ST22 clone has appeared in China and showed a trend to replace the predominant ST59 clone in some areas (20 - 23). Nevertheless, these studies are mainly based on epidemiological investigations without an in-depth analysis of the virulence characteristics and genomic features.

In this study, we collected 30 ST22 strains from Hubei, Sichuan, Zhejiang, Guangdong, Inner Mongolia Autonomous Region, and Shanghai in China. We conducted genomic and phenotypic characterization of these strains and reconstructed the molecular evolution of ST22 MRSA in China. Our results revealed that the ST22-MRSA-Vb clone in China, independent of the EMRSA-15 clone, likely evolved from native MSSA, while other ST22-MRSA-IV clones were imported from abroad. Importantly, we found that the ST22 SCC*mec*IVa-t309 MRSA strains, carrying both *pvl* and *tst* genes, have a higher virulence potential than other dominant clones in China (e.g., ST5, ST59, and ST239) and USA300 strain.

## MATERIALS AND METHODS

For full details, see SI Materials and Methods.

### Collection of *S. aureus* clinical strains

A total of 565 non-duplicated MRSA clinical strains were obtained from seven tertiary hospitals in seven provinces and municipalities in China, from Hubei, Sichuan, Zhejiang, Guangdong, Inner Mongolia Autonomous Region, Shanghai, and Jiangxi.

### Antimicrobial susceptibility testing

A total of 18 antimicrobial agents were tested for antimicrobial susceptibility of 30 ST22 (29 MRSA and 1 MSSA) strains.

### Whole-genome sequencing

A 2 × 150-base pair paired-end reads was used for sequencing on the Illumina NovaSeq platform. The raw data were filtered and *de novo* assembled into contigs by using CLC Genomics Workbench software (version 12.0; CLCbio). The molecular characterization was conducted using online tools.

### Phylogenetic analysis and Bayesian evolutionary analysis

A previously described method was used to infer time-scaled phylogeny of ST22 strains (24). In brief, Snippy v4.6.0 was used to identify core single-nucleotide polymorphisms for the ST22 genome and the BactDating R package was used to estimate node dates of ST22 strains. The recombination-corrected tree from Gubbins output and the isolation dates were used as the inputs in BactDating v1.1.

### Mouse skin abscess model

The mouse skin abscess model was performed as described previously (25). Wilcoxon tests or unpaired two-tailed Student’s *t*-tests were performed to analyze statistical significance.

### *Galleria mellonella* infection model

*G. mellonella* (220, 320 mg each) were divided into seven groups (MSSA-21, HA-MRSA ST5, HA-MRSA ST239, CA-MRSA ST59, MR506, USA300, and phosphate-buffered saline [PBS]) (*n* = 10 *G*. *mellonella* in each group). *G. mellonella* were injected in the right hind paw with 10 μL containing 3 × 10^8^ colony-forming unit live *S. aureus* bacterial suspension.

### Analysis of hemolytic activities

Lysis of erythrocytes tests were carried out as described before (25). The hemolytic activities were identified by adding 200 μL supernatant samples to 800 μL PBS solution containing 3% sterile rabbit red blood cells (RRBCs) and incubating at 37°C for 1 hour.

### Biofilm semi-quantitative assay

Biofilm semi-quantitative assays were performed as described before (25).

### Quantitative enzyme-linked immunosorbent assay (ELISA) for α-toxin

The α-toxin was detected by a staphylococcal α-toxin ELISA kit (Sigma-Aldrich, St. Louis, MO, USA). Overnight *S. aureus* cultures were diluted 1:200 into 4-mL TSB for an additional 24 hours at 37°C and adjusted to a same absorbance at OD_600_ (optical density at 600 nm) for reserve.

### Real-time fluorescence quantitative PCR (RT-qPCR)

The expressions of the *agrA* and *RNAIII* genes in ST22 strains were evaluated by RT-qPCR with *gyr*B as an internal control.

### Statistical analysis

Unpaired two-tailed Student’s *t*-tests and Wilcoxon tests were performed to analyze statistical significance. All data in this study were analyzed using GraphPad Prism 8.0.2, and the error bars in all graphs represented mean ± SD. *P*-values <0.05 were considered statistically significant.

## RESULTS

### Molecular characteristics of ST22 strains

**Fig 1:**
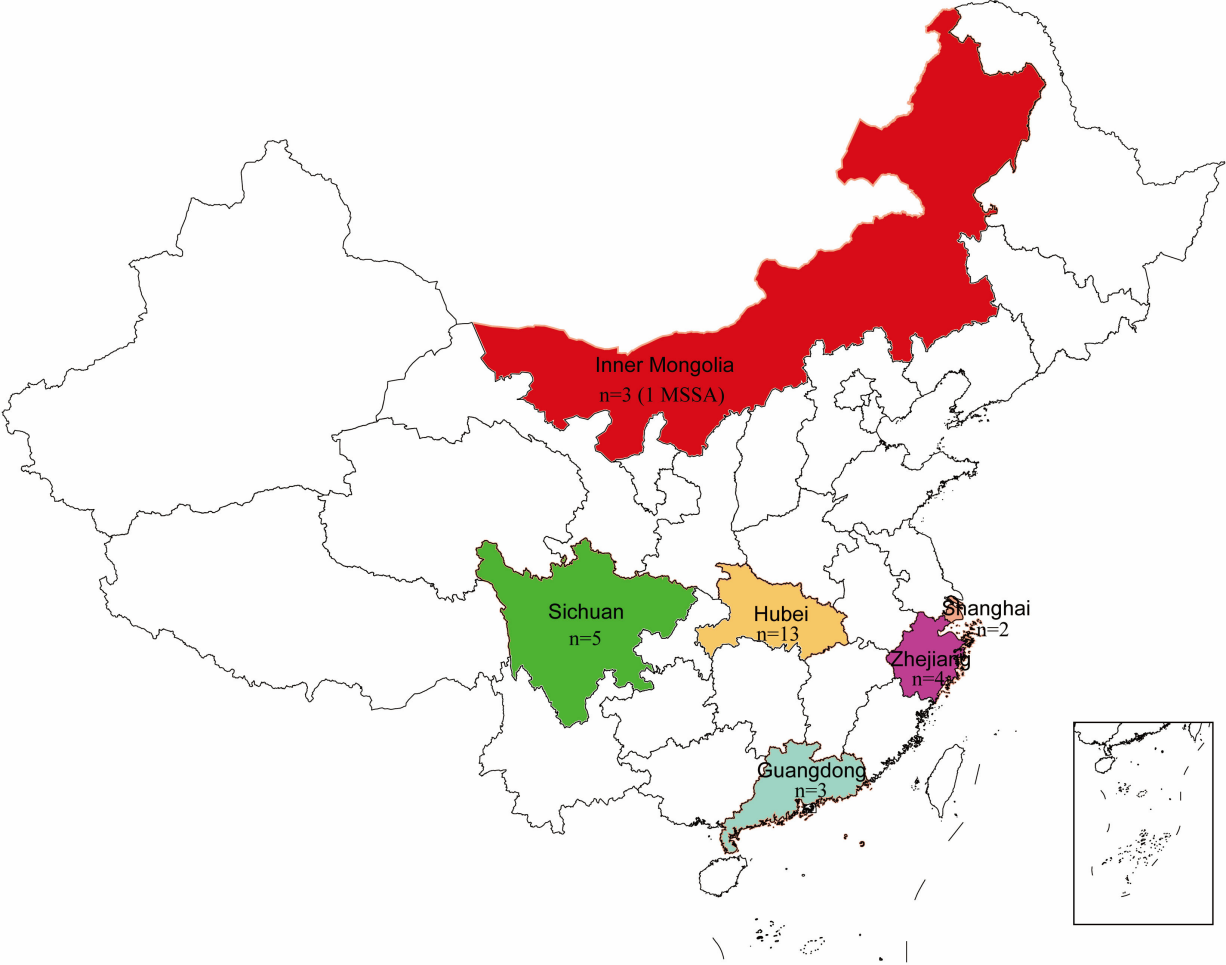
Geographical distribution of 30 ST22 clinical strains (29 MRSA strains and 1 MSSA strain) in this study.

A total of 29 non-duplicate ST22 MRSA strains from six tertiary hospitals distributed in six provinces in China were detected from 565 MRSA strains (Fig. 1). They were from Hubei (13 strains from September 2017 to July 2020), Sichuan (5 strains from January 2018 to June 2020), Zhejiang (4 strains from February 2019 to June 2020), Guangdong (3 strains from January 2017 to June 2019), Inner Mongolia Autonomous Region (2 strains from February 2016 to June 2020), and Shanghai (2 strains from January 2017 to June 2020). One additional ST22 MSSA strain was collected from the Mongolian Autonomous Region.

Among these 29 ST22 MRSA strains, 19 strains were isolated from pus and wound secretion specimens from patients with subcutaneous abscess, cellulitis, paronychia, and otitis media. The remaining 10 strains were from sputum (*n* = 6) and blood specimens (*n* = 4). The one ST22 MSSA strain collected from the Mongolian Autonomous Region was isolated from the wound secretion specimens.

The *spa* typing discriminated the 29 ST22 MRSA strains into five types, and one strain had a new *spa* type (Table 1). The *spa* t309 was the most frequently detected type, accounting for 72.4% (21/29), followed by *spa* t15183 (10.3%, 3/29) and *spa* t005 (6.9%, 2/29). Only one t790 and one t474 were detected (1/29). The *spa* type of the ST22 MSSA strain was also t309 as well.

**TABLE 1:** Characteristics of ST22 strains

SCC*mec* (no.)	*spa* types (no.)	Regions (no.)	Specimen source (no.)	*lukS/lukF* (y/n)^a^
IVa (14)	t309 (12)	Hubei (12)	Pus and wound secretions (11) Blood (1)	y (12)
	t005 (2)	Inner Mongolia Autonomous Region (2)	Pus and wound secretions (2)	y (2)
IVh (3)	t15183 (3)	Guangdong (3)	Sputum (3)	n (3)
IVc (1)	t474 (1)	Zhejiang (1)	Sputum (1)	y (1)
IVd (1)	t790 (1)	Zhejiang (1)	Pus and wound secretions (1)	n (1)
Vb (10)	t309 (9)	Sichuan (5), Shanghai (2), Hubei (1), Zhejiang (1)	Pus and wound secretions (5) Blood (3) Sputum (1)	y (7) n (2)
	New (1)	Zhejiang (1)	Sputum (1)	y (1)

^*a*^ y, *lukS*/*lukF* gene was positive; n, *lukS*/*lukF* gene was negative.

Two SCC*mec* types (IV and V) were identified among the 29 MRSA strains. The majority of MRSA strains belonged to SCC*mec* IV (65.5%, 19/29), and the others were SCC*mec* V (34.5%, 10/29). SCC*mec* IV is further divided into SCC*mec* IVa (48.3%, 14/29), SCC*mec* IVh (10.3%, 3/29), SCC*mec* IVc (3.4%, 1/29), and SCC*mec* IVd (3.4%, 1/29), while SCC*mec* V strains were all SCC*mec* Vb (34.5%, 10/29). ST22 MRSA strains in our study were dominated by the ST22 SCC*mec*IVa-t309 (41.4%, 12/29).

### Virulence factor–encoding genes and antimicrobial resistance genes in ST22 strains

**Fig 2:**
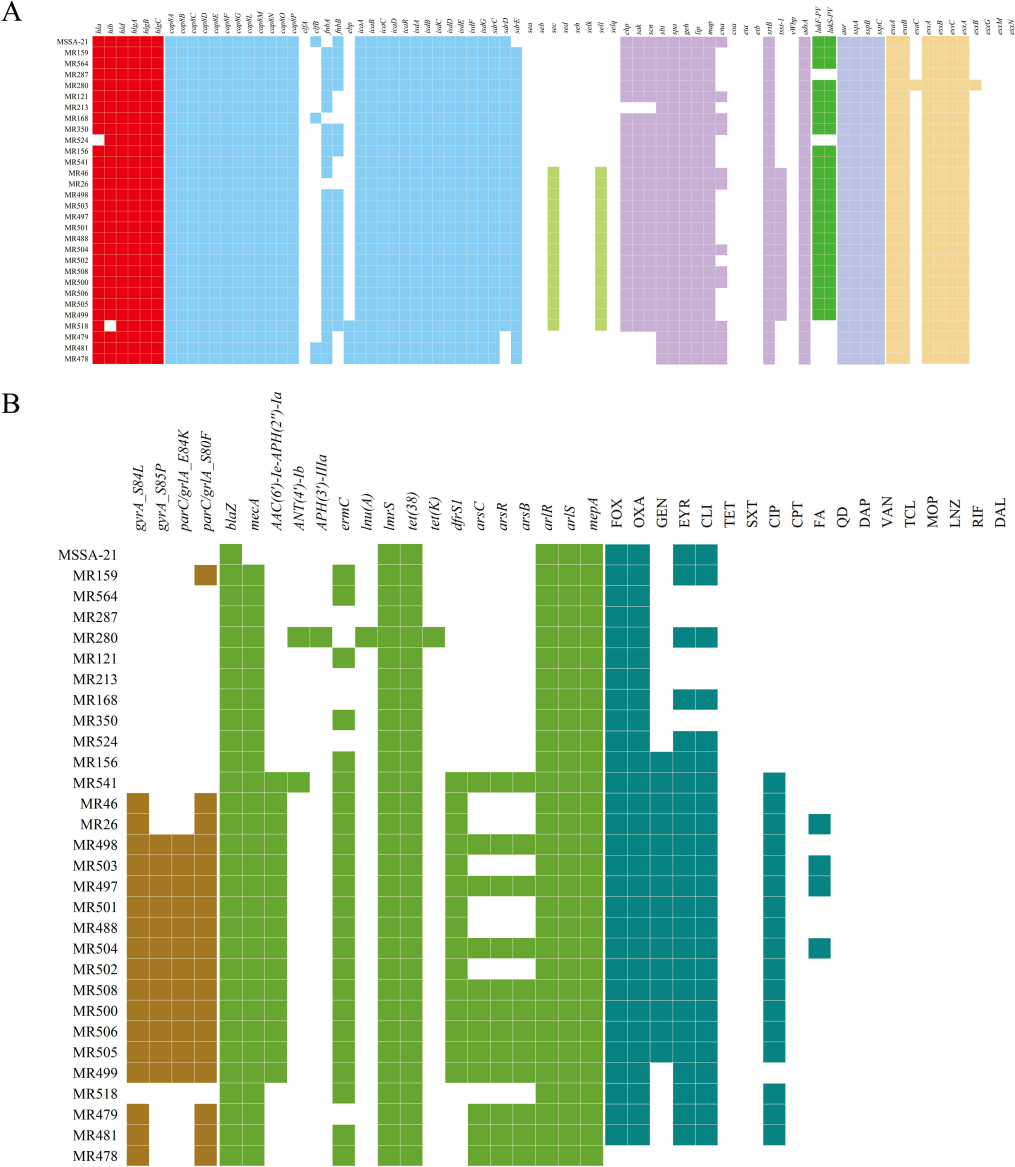
The distribution of virulence genes and antimicrobial resistance genes of 30 ST22 *S. aureus* strains in this study. (**A**) The heatmap of virulence genes across the 30 ST22 *S. aureus* strains. White blocks represent the absence of genes, and colored blocks represent the presence of genes. (**B**) The heatmap of mutations, antimicrobial-resistance genes, and antibiotic susceptibility profiles across the 30 ST22 *S. aureus* strains. White blocks represent the absence of genes or susceptibility to antibiotics, and colored blocks represent the presence of genes or resistance to antibiotics.

The different distributions of the virulence factor–encoding genes are shown in Fig. 2A. We found that the presence of most virulence genes in ST22 strains was similar and only a few genes were distributed differently. For example, almost all strains carried hemolysin-associated *hla*, *hlb*, *hld*, *hlgA*, *hlgB*, and *hlgC* genes, which have been shown to make an important impact on skin colonization and infection. Only the SCC*mec* IVa strains and the SCC*mec* IVd strain MR518 harbored *sec* and *sel* genes encoding enterotoxins. The toxic shock syndrome gene *tst* also existed only in the ST22 SCC*mec*IVa strains. Furthermore, 24 strains were positive for the Panton–Valentine leucocidin (PVL) genes *lukS-PV* and *lukF-PV*. Twenty-six strains were found harboring *chp*, *sak*, and *scn* genes. These genes were found in the φSaint3 β-hemolysin-converting bacteriophages, and a previous study showed that they contribute to potent immune evasion during host defense against *S. aureus* infections, associated with increased virulence in ST22 (26, 27).

We then examined the presence of antibiotic-resistance genes in our ST22 strains. The presence or absence of quinolone resistance–determining regions (QRDRs) mutations (left), anti-microbial resistance (AMR) genes (middle), and antibiotic-resistance profiles (right) for each strain is shown in Fig. 2B. Mutations associated with quinolone resistance in *S. aureus* were commonly found in ST22 strains, with 56.7% (17/30) presenting the double mutation *gyrA*_S84L and *parC*_S80F in the QRDRs. In addition, there was a single strain with a point mutation of *parC*_S80F. The *in vitro* antimicrobial susceptibility results of the 30 strains were in accordance with genomic analysis results, and most strains were multidrug resistant (MDR) (80.0%) (Table S1). It is worth mentioning that all ST22 SCC*mec*IVh strains were resistant to ciprofloxacin and erythromycin but susceptible to gentamicin and trimethoprim–sulfamethoxazole, which was consistent with a typical ST22-IV antibacterial spectrum (28). Likewise, all ST22 SCC*mec*IVa strains were resistant to ciprofloxacin, gentamicin, erythromycin, and clindamycin.

### General characterization and phylogenetic construction of global ST22 clones

**Fig 3:**
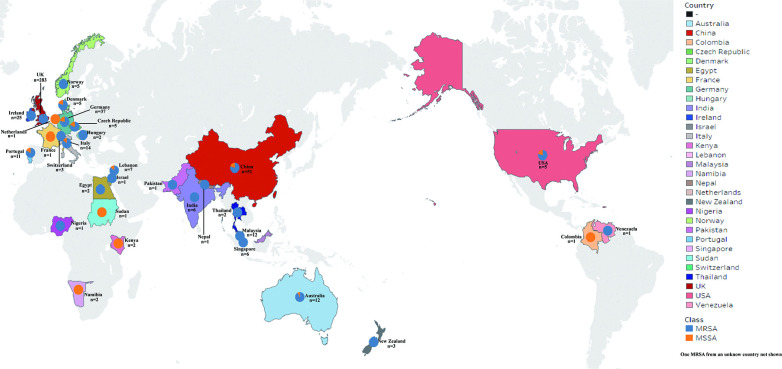
Geographical distribution of 510 ST22 *S. aureus* strains.

Among the 510 *S. aureus* ST22 strains, 51 were from China (30 ST22 strains from our collection and 21 from a previous study [29]), and the others were isolated from Britain, Germany, Italy, Australia, Malaysia, Portugal, and other countries(Fig. 3). BactDating estimates that the most recent common ancestor (MRCA) of global ST22 strains was around 1951 (95% CI 1942 to 1958).

**Fig 4:**
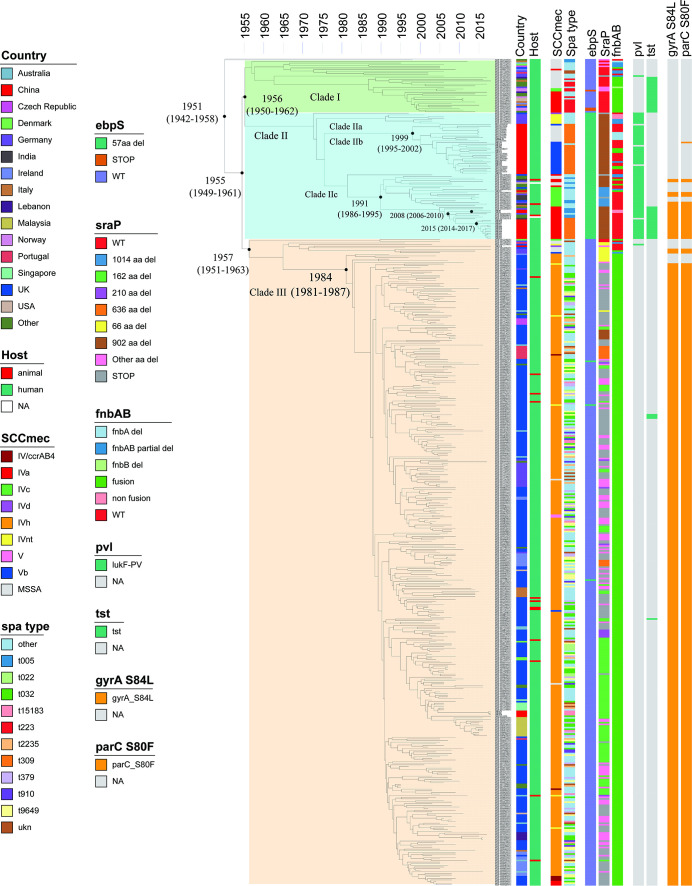
Phylogenetic analysis of 510 genomes of ST22 strains. Colors in columns illustrated country, host, SCC*mec*, *spa* type, *ebpS*, *Srap*, *fnbAB*, *pvl*, *tst* genes, and the QRDR mutations (*gyrA* and *parC*). Selected divergence time and 95% CIs are shown at the nodes.

Our analysis divided the 510 ST22 genomes into three main clades (I, II, and III), and the separation time of the three clades was from about 1955 (95% CI 1949 to 1961) (Fig. 4). The ancestor of clade I (*n* = 33) and clade II (*n* = 78) originated around 1956 (95% CI 1950 to 1962). The hosts of clade I were all humans, while clade II also included animals (3.8%). In clade I, approximately half of the strains (17/33) were MSSA, belonging to diverse *spa* types: t223, t005, and others. Most of the remaining ST22 MRSA strains (14/16) harbored SCC*mec* IVa. Notably, clade I was associated with a high prevalence of toxic shock syndrome toxin (*tst*) gene while only one ST22 MSSA strain carried Panton–Valentine leucocidin (*pvl*).

The clade II can be divided into three subclades (IIa, IIb, and IIc) and two singletons. The subclade IIa included strains from United Kingdom and Germany (belonging to SCC*mec* IVd-t474 and SCC*mec* IVnt-t474, respectively). The subclade IIb emerged in ~1999 (95% CI 1995 to 2002) were all from China (*n* = 31), and the MRSA strains of this subclade all harbored SCC*mec* Vb. Except for one MRSA strain whose *spa* type was t474, the rest of the strains in this subclade belonged to t309. The subclade IIc is the largest clade II subclade (*n* = 39) and emerged in ~1982. The majority of strains were collected in China, United Kingdom, Germany, India, Italy, and other countries. Subtypes IVa (23/39) and IVc (12/39) SCC*mec* cassettes were prevalent in MRSA strains (35/39) in this subclade with *spa* types identified as t309, t005, and the others. The presence of the *pvl* (present in 94.9% of genomes) was a characteristic feature of clade II, while the *tst* was absent in 82.1% of genomes.

The clade III was represented by highly similar EMRSA-15 strains, and the EMRSA-15 clade mainly consisted of strains from the United Kingdom, but also from other European countries such as Germany and Ireland, as well as Asian countries such as Singapore and Malaysia. Almost all of the strains of this clade carried type IVh SCC*mec*, with other types IV (e.g., IVa, IVc, IVd), Vb, and V found in a few strains. Unlike clade I and II, *pvl* and *tst* genes were almost absent in clade III.

In addition to the differences in the presence of *pvl* and *tst* genes between ST22 strains in China and ST22 strains from other countries, the three clades also differed in the amino acid sequences of elastin-binding protein S (*ebpS*) genes, serine-rich adhesin for platelets (*SraP*) genes and fibronectin-binding protein (*fnb*) genes. *ebpS* and *SraP* in clade I strains were almost wild type, while *fnb* was almost *fnbA–fnbB* gene fusion. All *ebpS* of clade II strains were 57 aa del, most *SraP* had 902 aa del, and 11 strains had additional 102 aa deletion in other fragments on the top of 902 aa deletion. In addition, the *fnb* of this clade displayed gene variations. Almost all *ebpS* in clade III strains were wild type, and *fnb* was almost *fnbA–fnbB* gene fusion. However, *SraP* of this clade was characterized by a significant variation, including 162 aa deletion, 66 aa deletion, 902 aa deletion, 636 aa deletion, and so on.

Among the 30 ST22 strains we collected in China, 26 strains (ST22-t039-SCC*mec* IVa/Vb, ST22-t005-SCC*mec* IVa, ST22-t474-SCC*mec* IVc, and ST22-new-SCC*mec* Vb) belonged to subclade IIb (11/26) or subclade IIc (15/26), while 4 strains (ST22-t15183-SCC*mec* IVh and ST22-t790-SCC*mec* IVd) to clade III (EMRSA-15), indicating that the Chinese ST22 lineages were not derived from the same origin. The previous study by Zhou et al. collected 21 ST22 strains (9 MSSA and 12 MRSA, respectively) and almost all strains (20/21) belonged to subclade IIb (29). All subclade IIb MRSA strains (11 MRSA from their study and 10 MRSA from this study) were phylogenetically different from ST22-SCC*mec* Vb clones in other regions. In this subclade, *spa* types almost all belonged to t309. Closely related MSSA and MRSA strains were also identified, demonstrating the regional transmission of ST22 MSSA and MRSA in China. The results suggested that SCC*mec* Vb ST22 in China likely originated by *spa* t309 MSSA strains independently acquired the SCC*mec* Vb cassette.

Interestingly, different from the previous ST22 study in China (29), we identified 15 ST22 MRSA strains belonging to a novel subclade IIc. These 15 strains including harbored subtypes IVa (14/15) and IVc (1/15) SCC*mec* cassettes with *spa* types t309 (12/15), t005(2/15), or t474 (1/15) were collected from Hubei, Inner Mongolia Autonomous Region, and Zhejiang. Noteworthy, 14 strains from our collection in subclade IIc had both *pvl* and *tst* genes.

Five ST22 MRSA strains (four ST22-IVh and one ST22-IVd, respectively) from China were found in clade III. They clustered in two distinct subclades, with different SCC*mec* elements and *spa* types. Among them, three ST22-t15183-SCC*mec* IVh (this study) and one ST22-t032-SCC*mec* IVh strains were clustered with strains from Singapore and Malaysia, and the strain of ST22-t790-SCC*mec* IVd we collected was closely related with strains from the United Kingdom. Unlike the other 4 ST22-IVh strains, the ST22-IVd strain possessed the *pvl*, with the *sraP* 72 aa deletion and wild-type *fnb*.

We then evaluated the presence of QRDR mutations linked to fluoroquinolone resistance. There were significant differences among clades I–III. Both *gyrA* and *parC* genes in clade I were wild type, and the mutations in the *gyrA* and *parC* genes (*gyrA*_S84L and *parC*_S80F) in clade II were mainly concentrated in IIc, whereas mutations (*gyrA*_S84L and *parC*_S80F) were present in most strains of clade III.

### ST22 strains displayed high hemolytic capacity and low biofilm-forming ability *in vitro*

Erythrocyte lysis capacity assays and semi-quantitative biofilm formation tests were performed on all 30 ST22 strains (Fig. S1A and B). The hemolytic ability of most strains was stronger than that of USA300, while the biofilm-forming ability was weaker than that of USA300. When the strains were divided by clades, we found that the three clades exhibited comparable erythrocyte lytic capacity, with no statistical differences. There was no significant difference between subclade IIb and subclade IIc in biofilm formation ability. In contrast, clade III exhibited a higher biofilm-forming ability compared to the other two clades. The differences were significant between clade III and subclade IIb or between clade III and subclade IIc (*P* < 0.001). Notably, the three EMRSA-15-like clade III strains exhibited both high erythrocyte lysis capacity and high biofilm formation ability.

### Virulence of different evolutionary subclade of the *S. aureus* ST22 strains

**Fig 5:**
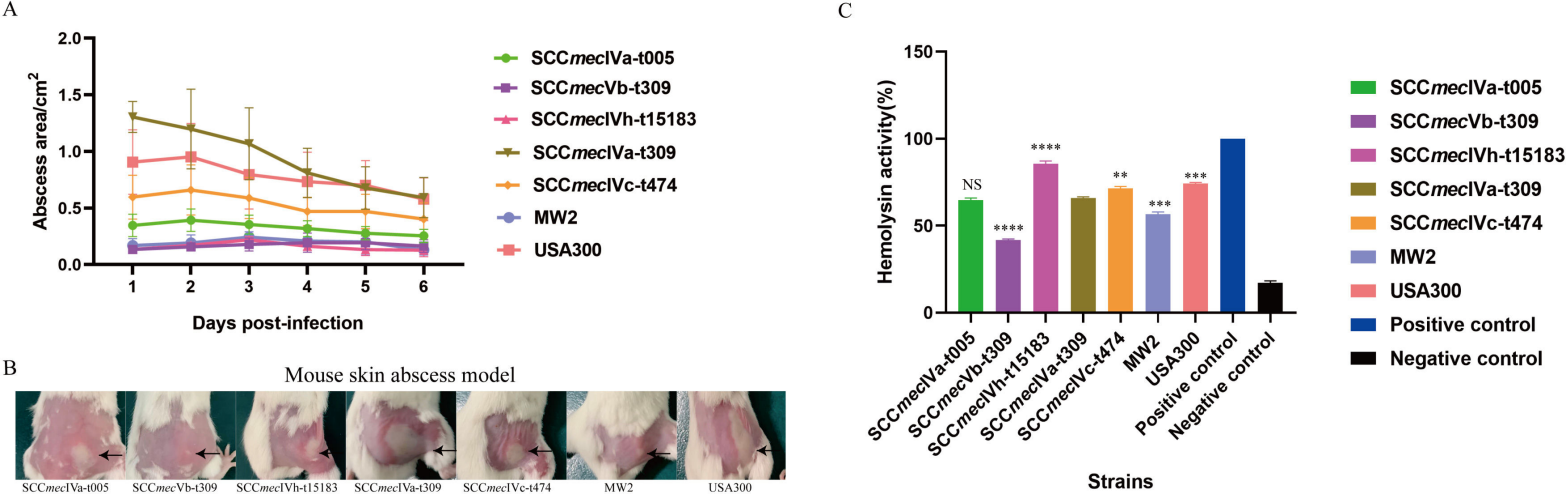
The mouse skin abscess infections caused by the selected *S. aureus* ST22 strains. (**A**) Graph of daily change in abscess size during skin abscess infection in mice. Five mice were infected per strain. (**B**) The abscesses caused by the selected *S. aureus* ST22 strains. The lesion size of abscess on the first-day post-infection for one representative mouse in each group was shown. (**C**) Analysis of hemolytic activities. The A_600_ absorbance value of the positive control value was 100%, and the absorbance measured at 600 nm for each sample was converted to the corresponding percentage. ^**^ *P* < 0.01; ^***^ *P* < 0.001, and ^****^ *P* < 0.0001; NS, not significant *P* ≥ 0.05.

We then randomly selected five strains from different evolutionary subclades to evaluate their virulence using an *in vivo* mouse skin abscess model. USA300 and MW2 (USA400), the virulent CA-MRSA strains from the USA were used as control strains. The seven strains showed different abilities to cause skin abscesses in mice (Fig. 5A). Notably, the SCC*mec*IVa-t309 strain (subclade IIc), which was the most common ST22 MRSA genotype in our collection, caused the most severe abscesses, with comparable abscess sizes to USA300 (*P* = 0.075). The remaining strains caused abscesses with a smaller size than USA300 (*P*< 0.001), but significantly larger (*P* < 0.0001) or comparable in size compared with MW2. In addition, we compared the lysis of RRBCs of these strains and found that the hemolytic capacity was also quite different (Fig. 5C), and EMRSA-15-like clade III SCC*mec*IVh-t15183 strain displayed the highest hemolytic capacity.

### Virulence of SCC*mec*IVa-t309 strains *in vitro* and *in vivo*

By comparing the virulence of ST22 strains in different evolutionary subclades in the mouse skin abscess model, we recognized that the virulence levels of ST22 strains were quite different, and the predominant ST22 SCC*mec*IVa-t309 strains have the highest virulence potential. To further understand the virulence differences between ST22 SCC*mec*IVa-t309 strain with other common China HA-MRSA (ST5, ST239, respectively) and CA-MRSA (ST59) strains, and the USA300 strains, we firstly selected four strains (one of each of ST5, ST239, ST59, and USA300) of different clones and compare their *in vitro* and *in vivo* virulence with SCC*mec*IVa-t309 strain MR506. The MSSA ST22 strain MSSA-21 was also included for comparison, as previous studies showed *mec*A may have an inhibitory effect on virulence (30, 31).

**Fig 6:**
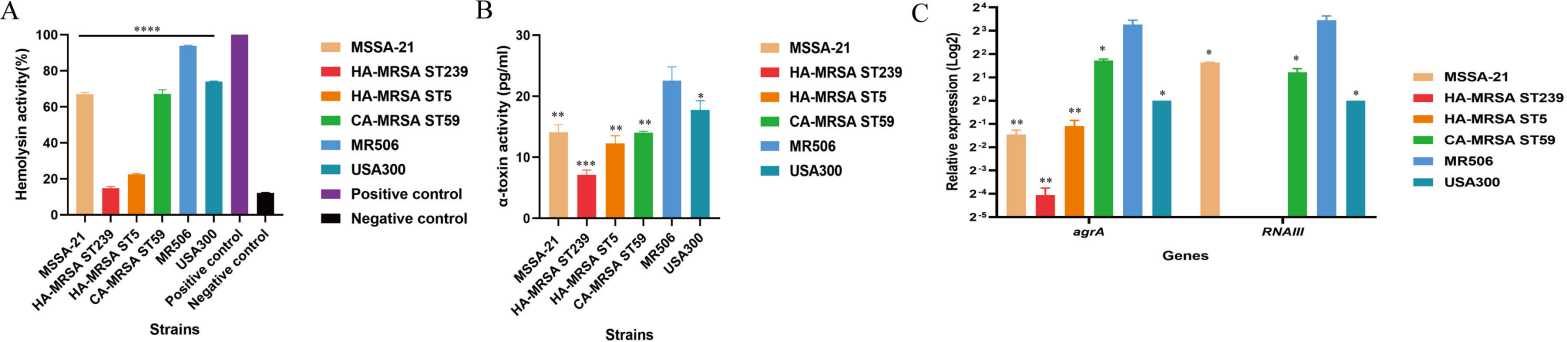
Hemolysis ability of MR506 strain compared with ST22 MSSA-21 strain, HA-MRSA strains (ST239 and ST5), CA-MRSA ST59 strain, and USA300. (**A**) Analysis of hemolytic activities. The A_600_ absorbance value of the positive control value was 100%, and the absorbance measured at 600 nm for each sample was converted to the corresponding percentage. (**B**) The α-toxin expression of selected strains quantified by ELISA. (**C**) Relative expressions of *agrA* and *RNAIII* in *S. aureus* ST22 strains and comparative strains. ^*^ *P* < 0.05; ^**^ *P* < 0.01; ^***^ *P* < 0.001; ^****^ *P* < 0.0001.

Hemolysis test (red blood cell lysis), the α-toxin production level, and RT-qPCR were performed. As shown in Fig. 6A and B, the MR506 had the strongest hemolytic activity in comparison to the other strains, suggesting that MR506 may have a higher virulence potential than other common HA-MRSA and CA-MRSA strains in China (*P* < 0.0001). In addition, the hemolytic activity of MR506 was also stronger than that of MSSA-21, indicating that the acquisition of *mec*A may not comprise the virulence in ST22 strains. The α-toxin production level of MR506 was much higher than those of the other strains, which was consistent with the results of hemolytic activity. We then performed RT-qPCR to evaluate the expression of *agrA* and *RNAIII*. The mRNA levels of *agrA* and *RNAIII* were highly expressed in MR506 compared with MSSA-21, HA-MRSA (ST5, ST239), CA-MRSA (ST59), and the USA300 strains (*P* < 0.05) (Fig. 6C).

**Fig 7:**
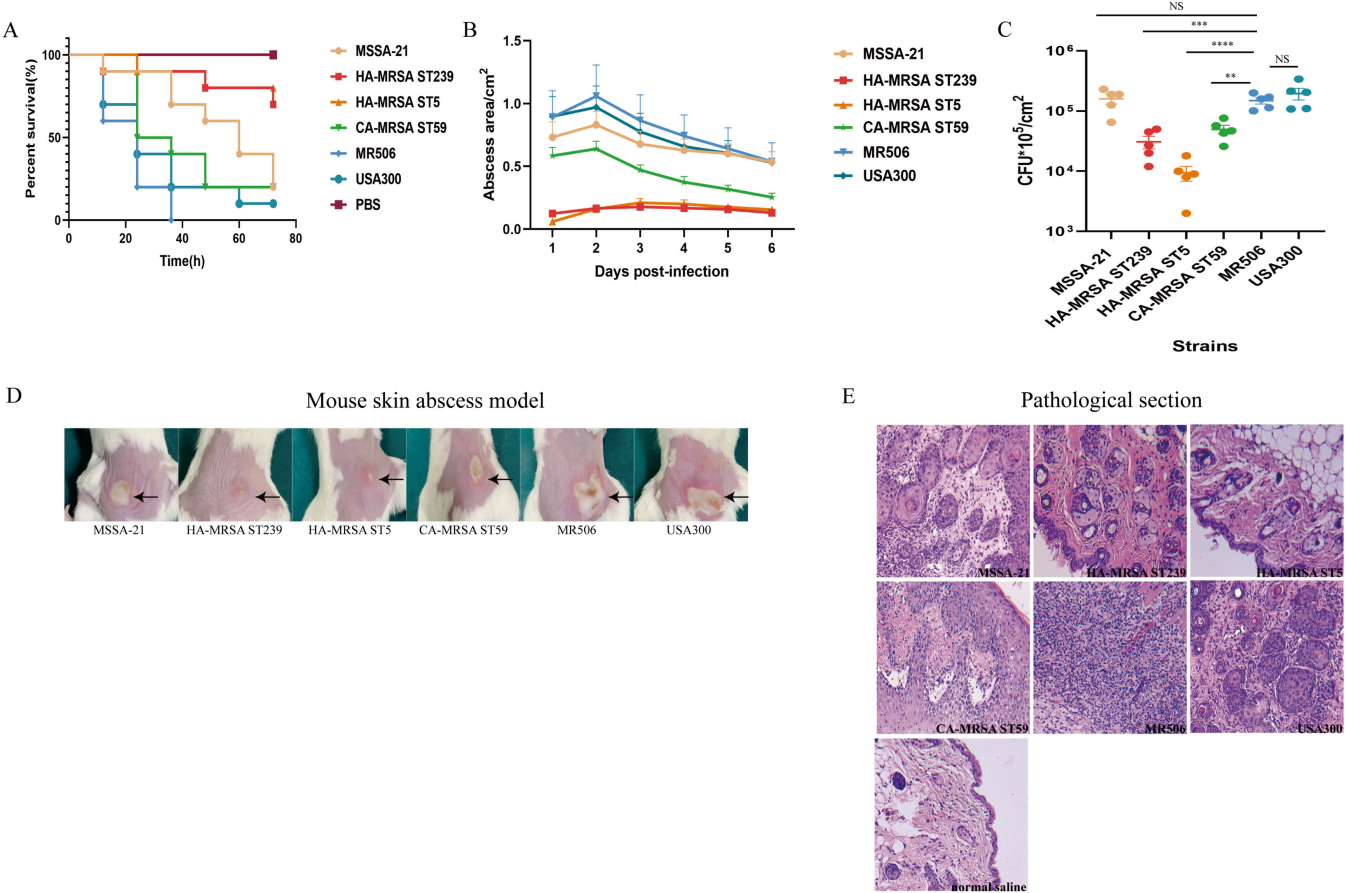
The *G. mellonella* infection model and mouse skin abscess infection model caused by ST22 SCC*mec*IV-t309 MR506 strain compared with ST22 MSSA-21 strain, HA-MRSA strains (ST239 and ST5), CA-MRSA ST59 strain, and USA300 strain. (**A**) The survival rates of the *G. mellonella* after infection with selected strains. (**B**) Graph of daily change in abscess size during skin abscess infection in mice. Five mice were infected per strain. (**C**) Bacterial load in skin abscesses in mice infected with selected strains. Five mice were infected per strain. (**D**) The lesion size of abscess on the second-day post-infection for one representative mouse in each group was shown. (**E**) Representative pathological sections of mouse skin abscesses from each group. ^**^ *P* < 0.01; ^***^ *P* < 0.001; ^****^ *P* < 0.0001; NS, not significant *P* ≥ 0.05.

To evaluate the potential virulence of the MRSA strain (MR506) *in vivo*, the *G. mellonella* infection and mouse skin abscess models were used. As shown in Fig. 7A, the *G. mellonella* infected with MR506 were all died within 36 hours (*n* = 10). The survival rate of *G. mellonella* was significantly lower following the infection with MR506 as compared to HA-MRSA ST5 and HA-MRSA ST239 strains (*P* < 0.0001). In addition, the survival rates of USA300, MSSA-21, and CA-MRSA ST59 strains were similar, but all were higher than that of MR506.

In mouse skin abscess models (Fig. 7B), the sizes of skin abscesses in mice infected with MR506 were comparable to that of USA300 and MSSA-21 (*P* > 0.05). Compared with MR506, the skin abscess sizes of mice infected with CA-MRSA ST59 (*P* < 0.01) and HA-MRSA strains (ST5 and ST239) (*P* < 0.0001) were significantly smaller, especially for the two HA-MRSA strains.

Bacterial burden was then accessed in the abscessed skin of infected mice (Fig. 7C). The burdens of skin abscesses in mice infected with MSSA-21, MR506, and USA300 strains were not statistically significant on the sixth day after infection. However, the burdens in the skin abscesses of mice of the HA-MRSA ST239 (*P* < 0.001) and CA-MRSA ST59 (*P* < 0.01) groups were lower than those of MSSA-21, MR506, and USA300 strains, while the burden of HA-MRSA ST5 (*P* < 0.0001) group was the lowest.

Histopathology of skin abscesses was performed on mice challenged with different strains. Histopathological sections showed that all six strains caused inflammatory infiltration in the epidermis (Fig. 7E). The skin abscesses of mice infected with the MR506 presented noteworthy inflammatory infiltrate. Inflammatory infiltration was similar between MR506 and USA300, with minimal inflammatory infiltration in ST5. These findings suggested that the MR506 strain was highly virulent, causing strong infections of the skin and capable of skin invasion.

**Fig 8:**
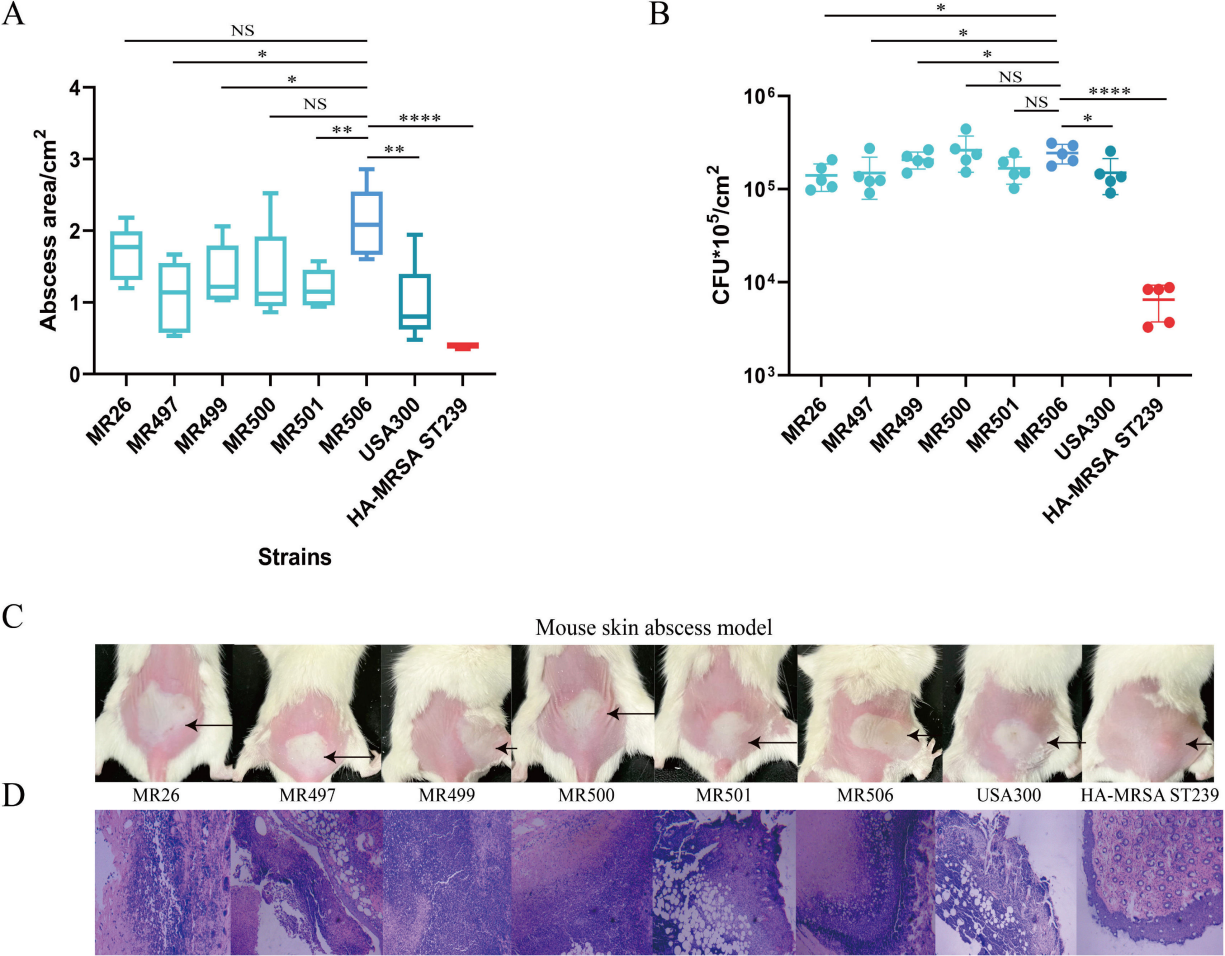
The mouse skin abscess infection model caused by randomly selected five strains from clade IIc compared with MR506, USA300 strain, and HA-MRSA ST239. (**A**) Back skin abscess areas of 40 mice (5 mice for each group) using the formula L × W after 24-hour infection. (**B**) Bacterial load in skin abscesses in mice infected with selected strains (five mice for each group). (**C**) Representative mouse back skin abscess after 24-hour inoculation of selected strains. (**D**) Representative pathological sections of mouse skin abscess from each group. ^*^P < 0.05; ^**^ *P* < 0.01; ^****^ *P* < 0.0001; NS, not significant *P* ≥ 0.05.

To rule out that the enhanced virulence in MR506 is strain specific, we further randomly selected five strains from subclade IIc and compared the virulence with MR506, USA300, and the dominant Chinese clone HA-MRSA ST239 strains using the mouse skin abscess experiment (Fig. 8A through D). We found that the virulence levels of the five subclade IIc strains were comparable to or slightly weaker than MR506, and comparable to or slightly stronger than USA300, but were significantly stronger than that of HA-MRSA ST239. The study suggested that increased virulence in subclade IIc strains was not strain specific but was more likely associated with this clade.

## DISCUSSION

ST22 strains, especially EMRSA-15, emerged in the United Kingdom and have spread to many countries. In China, ST22 MRSA was only sporadically detected, though it showed the tendency to cause local spread in some regions. The genomic characteristics and evolutionary history of ST22-MRSA in China remain to be fully determined. In this study, we genomically and phenotypically characterized 30 ST22 strains collected from six provinces in China, most of which were isolated from pus and wound secretions.

The main antimicrobial susceptibility feature of EMRSA-15 (SCC*mec*IVh) is resistant to fluoroquinolones and macrolides and rarely to aminoglycosides (28, 32). However, in this study, we found that the clade II China ST22 strains were resistant to gentamicin, especially those strains harboring SCC*mec*IVa. We observed that *aac(6')-Ie/aph(2'')-Ia* was possessed by SCC*mec*IVa and SCC*mec*IVc strains, whereas the QRDR mutations in the *gyrA* and *parC* genes were present in all SCC*mec*IVa and SCC*mec*IVh strains. Phylogenetic reconstruction and time estimation suggested that the resistance to fluoroquinolones via *gyrA* and *parC* mutations emerged around ~1990s, which coincided with the introduction of fluoroquinolones into routine clinical practice in 1987 (33). It should be emphasized that the predicted genotypes (resistance to fluoroquinolones and aminoglycosides) of these strains were consistent with the results of antimicrobial susceptibility testing. Four SCC*mec*IVa-t309 strains also developed resistance to fusidic acid. These findings warn of the growing number of MDR ST22 MRSA strains.

Phylogenetic reconstruction of global ST22 strains detected three major clades (I–III), which was consistent with previous studies of Gostev et al. (34). Our clade I correlated with cluster C in the Gostev study, which was represented by the “GAZA clone,” characterized by SCC*mec* IVa-t223 and high prevalence of *tst*. Despite being found in Gaza, Russia, and some other countries, the clade I strains were not detected in China. Our clade II was consistent with cluster B in the Gostev study, which was heterogeneous and included MSSA and MRSA from different regions and of different *spa* and SCC*mec* types. Our clade III was mainly represented by the EMRSA-15 strains (cluster A in the Gostev study).

Our 30 ST22 strains belonged to clades II (IIb, *n* = 11; IIc, *n* = 15) and III (*n* = 4), and our results showed that the “China clone” consisted of distinct lineages generated by independent acquisition of distinct SCC*mec* cassettes. Clade II showed high genetic diversity, despite that they were all evolved from the same ancestor. For example, clade II strains acquired SCC*mec* V (IIb), while IIc had multiple independent acquisitions of different SCC*mec* in the evolution process. Phylogenetic reconstruction and time estimation suggested that the ancestor of ST22 China clones emerged around 2001. ST22-SCC*mec*Vb strains may evolve from the native ST22-MSSA, and the China ST22-MRSA-Vb clones were unrelated to the epidemic EMRSA-15 strains. Other ST22-SCC*mec*IV strains (t309-SCC*mec*IVa, t005-SCC*mec*IVa, t474-SCC*mec*IVc, and t15183-SCC*mec*IVh) were closely related to strains from United Kingdom, Germany, Singapore, Malaysia, and other countries, suggesting that these strains may spread from other countries to China. This study further deepened the understanding of the evolution of ST22 in China.

*S. aureus* produces a variety of virulence molecules, including the most prominent pore-forming toxins Hla, PVL, and the adhesion-related FnBPA or FnBPB, which are known to be involved in invasiveness, causing severe skin and soft infections, but occasionally with severe necrotizing pneumonia and sepsis (35 - 38). In this study, 96.7% of clinical MRSA strains carried the *hla* gene and most strains have strong hemolytic activity. Meanwhile, 80% were PVL-positive and the PVL-positive strains may consequently contribute to increased inflammation, abscess formation, and tissue necrosis. Except for PVL, MRSA strains with TSST are likely to cause more complicated infections. In this study, we found that *pvl* (*lukS-PV* and *lukF-PV*) and *tst* genes co-existed in 14 subclade IIc strains whose genotypes were SCC*mec*IVa-t309 or SCC*mec*IVa-t005. In general, *S. aureus* strains rarely harbor both *pvl* and the *tst* genes (39). The co-existence of *pvl* and *tst* genes may indicate hypervirulence. Adhesion genes such as elastin-binding protein (*ebp*) and fibronectin-binding protein (*fnbA* and *fnbB*) are responsible for *S. aureus* adherence to epithelial cells. Among the strains we collected, we observed that only four clade III ST22 MRSA strains contain wild-type *ebp* genes, while the remaining *ebp* genes all had 57 aa deletion. Molecular studies have shown that both FnBPA and FnBPB are required for sepsis infection. In this study, the *fnbAB* of most of the strains were wild type, while six MRSA strains were shown to contain the *fnbA–fnbB* gene fusion. These surface components enable bacteria to adhere to various surfaces and form biofilms, making them resistant to various antimicrobial agents (40). Through biofilm semi-quantitative assay, we have found that strains with high biofilm-forming ability were present in clade III.

In the present study, we identified a novel subclade IIc ST22 strains, harboring both *pvl* and *tst*, which may also in part contribute to the high virulence. Compared with other common China HA-MRSA and CA-MRSA strains, interestingly, ST22 SCC*mec*IVa-t309 strains showed higher levels of lethal capacity, abscess formation, hemolysis, and α-toxin production than the other five comparative strains in *G. mellonella* infection model, mouse skin abscess model, and phenotypic tests (e.g., hemolysis activity and α-toxin quantification). The RT-qPCR on *agrA* and *RNAIII*, which regulate toxins most frequently associated with the virulence in ST22 strains, showed that the expressions of these genes in MR506 were higher than that of the comparison strains. This was consistent with the results of the hemolysis and alpha-toxin ELISA assays. The ST22 SCC*mec*IVa strains (subclade IIc) showed strong abscess-forming ability, even stronger than USA300, which has been notorious for its high virulence (41). These above results further indicated that ST22 SCC*mec*IVa-t309 strain had a higher virulence potential than other China common MRSA clones, and the increased virulence may be in part attributable to the elevated expression of virulence regulation genes, such as *agrA* and *RNAIII*. Additional molecular mechanisms underlying the high virulence potential in ST22 SCC*mec*IVa-t309 strain are undergoing further studies.

Collectively, we performed phenotypic and genetic characterization of ST22 MRSA strains in China and dissected their evolutionary relationships with global strains. Our results showed that China ST22 strains were not from the same clones. We found that the subclade IIb ST22-MRSA-Vb clone in China was independent of the EMRSA-15 clone, which appears to evolve from native ST22 MSSA strains through the acquisition of SCC*mec* in China. Notably, we also found a novel subclade IIc in China, which appeared to be imported from abroad. Among them, the ST22 SCC*mec*IVa clone carrying both *pvl* and *tst* and displayed significantly higher *in vitro* and *in vivo* virulence in comparison to other clade/subclade ST22 strains as well as other common China HA-MRSA or CA-MRSA strains. The further spread of these hypervirulent ST22 MRSA strains in other regions in China will likely lead to severe and hard-to-treat infections. Effective infection control and surveillance strategies should be developed to monitor and control their transmission.

## Data Availability

The raw reads of the 30 ST22 *Staphylococcus aureus* genomes sequenced in this study were deposited in GenBank under BioProject accession no. PRJNA929648.

## SUPPLEMENTAL MATERIAL

FIG S1 msystems.01242-22-s0001.tif (A) Erythrocyte lysis capacity (separated by 3 clades) and (B) semi-quantitative biofilm formation ability (separated by 3 clades) of the 30 ST22 S. aureus strains. The red line represented the reference strain USA300.

TABLE S1 msystems.01242-22-s0002.docx Antimicrobial susceptibility of 30 ST22 strains from China.

Supplementary Material 1 msystems.01242-22-s0003.pdf SI Materials and Methods.

ASM does not own the copyrights to Supplemental Material that may be linked to, or accessed through, an article. The authors have granted ASM a non-exclusive, world-wide license to publish the Supplemental Material files. Please contact the corresponding author directly for reuse.
