# Supplementary figures and images for: Phenotypic and genomic analysis of the hypervirulent ST22 methicillin-resistant Staphylococcus aureus in China
Source: mSystems. 2023 May 15;8(3):e01242-22. doi: 10.1128/msystems.01242-22 (PMC10308902; doi:10.1128/msystems.01242-22)

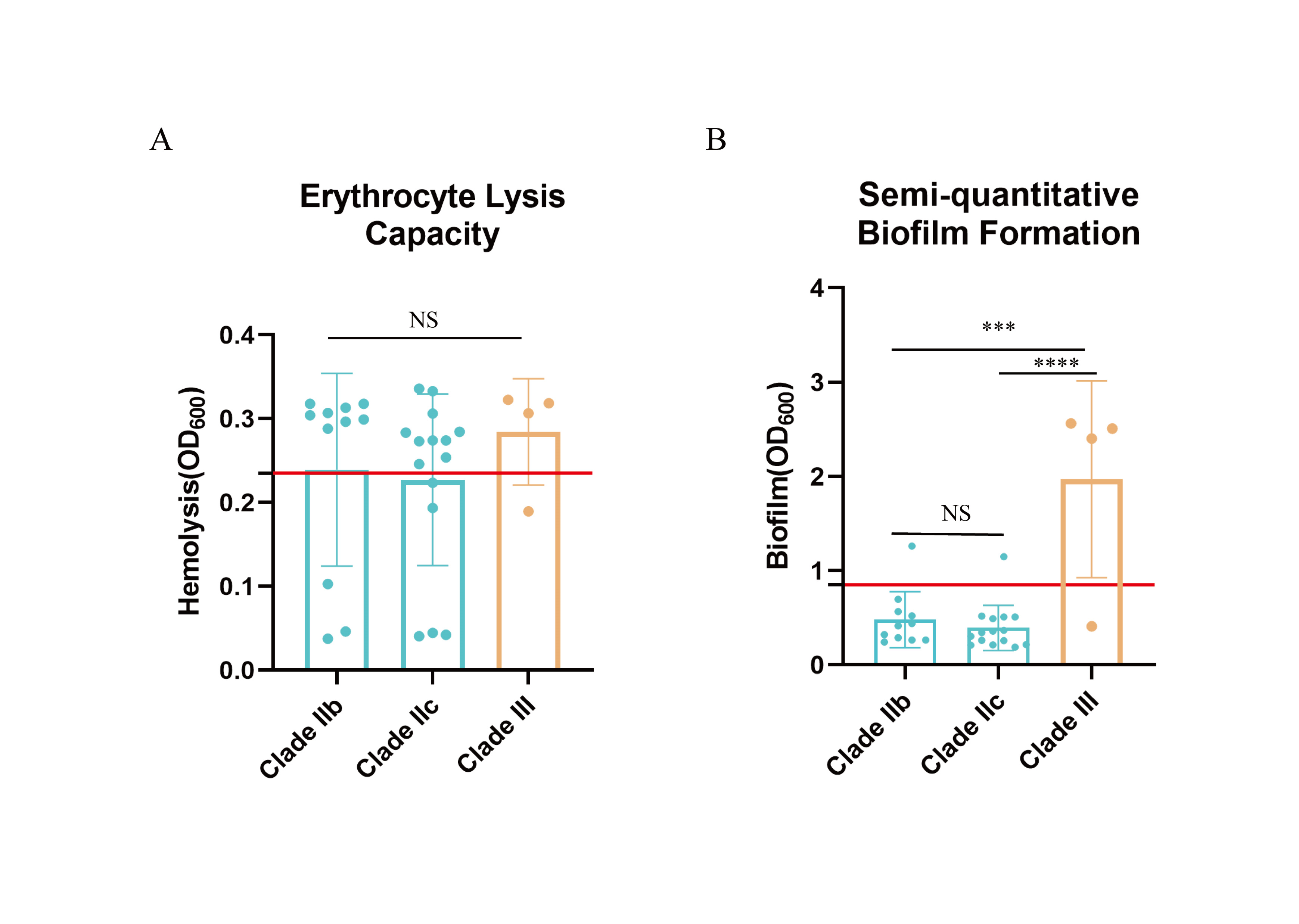

Supplement: FIG S1 — (A) Erythrocyte lysis capacity (separated by 3 clades) and (B) semi-quantitative biofilm formation ability (separated by 3 clades) of the 30 ST22 S. aureus strains. The red line represented the reference strain USA300. [file msystems.01242-22-s0001.tif]
