# Supplementary material for: Phenotypic and genomic analysis of the hypervirulent ST22 methicillin-resistant Staphylococcus aureus in China
Source: mSystems. 2023 May 15;8(3):e01242-22. doi: 10.1128/msystems.01242-22 (PMC10308902; doi:10.1128/msystems.01242-22)
Supplement: TABLE S1 — Antimicrobial susceptibility of 30 ST22 strains from China. [file msystems.01242-22-s0002.docx]

| Strains | CPT | CIP | EYR | CLI | TET | QD | FOX | OXA | DAP | VAN | GEN | FA | TCL | MOP | LNZ | RIF | DAL | SXT |
| --- | --- | --- | --- | --- | --- | --- | --- | --- | --- | --- | --- | --- | --- | --- | --- | --- | --- | --- |
| MSSA-21 | S | S | S | S | S | S | S | S | S | S | S | S | S | S | S | S | S | S |
| MR26 | S | R | R | R | S | S | R | R | S | S | R | S | S | S | S | S | S | S |
| MR46 | S | R | R | R(D) | S | S | R | R | S | S | R | S | S | S | S | S | S | S |
| MR121 | S | S | R | R(D) | S | S | R | R | S | S | S | S | S | S | S | S | S | S |
| MR156 | S | S | R | R(D) | S | S | R | R | S | S | S | S | S | S | S | S | S | S |
| MR159 | S | S | R | R(D) | S | S | R | R | S | S | S | S | S | S | S | S | S | S |
| MR168 | S | S | S | S | S | S | R | R | S | S | S | S | S | S | S | S | S | S |
| MR213 | S | S | S | S | S | S | R | R | S | S | S | S | S | S | S | S | S | S |
| MR280 | S | S | S | S | S | S | R | R | S | S | S | S | S | S | S | S | S | S |
| MR287 | S | S | S | S | S | S | R | R | S | S | S | S | S | S | S | S | S | S |
| MR350 | S | S | R | R(D) | S | S | R | R | S | S | S | S | S | S | S | S | S | S |
| MR478 | S | R | R | R(D) | S | S | R | R | S | S | S | S | S | S | S | S | S | S |
| MR479 | S | R | R | R(D) | S | S | R | R | S | S | S | S | S | S | S | S | S | S |
| MR481 | S | R | R | R(D) | S | S | R | R | S | S | S | S | S | S | S | S | S | S |
| MR488 | S | R | R | R(D) | S | S | R | R | S | S | R | S | S | S | S | S | S | S |
| MR497 | S | R | R | R(D) | S | S | R | R | S | S | R | R | S | S | S | S | S | S |
| MR498 | S | R | R | R(D) | S | S | R | R | S | S | R | R | S | S | S | S | S | S |
| MR499 | S | R | R | R(D) | S | S | R | R | S | S | R | S | S | S | S | S | S | S |
| MR500 | S | R | R | R(D) | S | S | R | R | S | S | R | S | S | S | S | S | S | S |
| MR501 | S | R | R | R(D) | S | S | R | R | S | S | R | R | S | S | S | S | S | S |
| MR502 | S | R | R | R(D) | S | S | R | R | S | S | R | R | S | S | S | S | S | S |
| MR503 | S | R | R | R(D) | S | S | R | R | S | S | R | S | S | S | S | S | S | S |
| MR504 | S | R | R | R(D) | S | S | R | R | S | S | R | S | S | S | S | S | S | S |
| MR505 | S | R | R | R(D) | S | S | R | R | S | S | R | S | S | S | S | S | S | S |
| MR506 | S | R | R | R(D) | S | S | R | R | S | S | R | S | S | S | S | S | S | S |
| MR508 | S | R | R | R(D) | S | S | R | R | S | S | R | S | S | S | S | S | S | S |
| MR518 | S | S | R | R | S | S | R | R | S | S | S | S | S | S | S | S | S | S |
| MR524 | S | S | S | S | S | S | R | R | S | S | S | S | S | S | S | S | S | S |
| MR541 | S | S | R | R(D) | S | S | R | R | S | S | R | S | S | S | S | S | S | S |
| MR564 | S | S | R | R(D) | S | S | R | R | S | S | S | S | S | S | S | S | S | S |
