# Supplementary material for: Phenotypic and genomic analysis of the hypervirulent ST22 methicillin-resistant Staphylococcus aureus in China
Source: mSystems. 2023 May 15;8(3):e01242-22. doi: 10.1128/msystems.01242-22 (PMC10308902; doi:10.1128/msystems.01242-22)
Supplement: Supplementary Material 1 — SI Materials and Methods. [file msystems.01242-22-s0003.pdf]

SI Materials and Methods

Collection of *S. aureus* clinical strains

A total of 565 non-duplicated MRSA clinical strains were obtained from seven tertiary hospitals in seven provinces and municipalities in China, from Hubei, Sichuan, Zhejiang, Guangdong, Inner Mongolia Autonomous Region, Shanghai and Jiangxi. These regions represent different levels of MRSA prevalence in China as described before(1). One additional ST22 MSSA clinical strain was collected from Inner Mongolia Autonomous Region. These MRSA clinical strains were re-confirmed with cefoxitin disk diffusion test and PCR targeting the *mec*A gene. The MRSA criteria were in accordance with the protocols provided by the Clinical and Laboratory Standards Institute (CLSI)(2).

Antimicrobial susceptibility testing

The disk diffusion method was used to test the antimicrobials susceptibility of 30 ST22 (29 MRSA and one MSSA) strains to ceftaroline (CPT), ciprofloxacin (CIP), erythromycin (EYR), clindamycin (CLI), tetracycline (TET), quinupristin-dalfopristin (QD) (Oxoid, UK) according the CLSI guideline. The D test was performed to determine the rate of erythromycin-induced resistance to clindamycin(2). Susceptibility to cefoxitin (FOX), oxacillin (OXA), daptomycin (DAP), vancomycin (VAN), gentamicin (GEN), fusidic acid (FA), teicoplanin (TCL), mupirocin (MOP), linezolid (LNZ), rifampicin (RIF), dalbavancin (DAL) and trimethoprim-sulfamethoxazole (SXT) (Sigma-Aldrich, United States) was assessed by microdilution in cation-adjusted Mueller-Hinton broth method to determine the minimun inhibitory concentrations (MICs). The MIC of fusidic acid was interpreted according to European Committee on Antimicrobial Susceptibility testing (EUCAST)(3). The results of other antibiotics were interpreted using the Clinical and Laboratory Standards Institute (CLSI)(2). S. aureus ATCC29213 and ATCC25923 strains were used as the quality controls.

Whole genome sequencing

The bacterial genomic DNA was extracted using UltraClean Microbial Kit (Qiagen, NW, Germany). A 2 × 150-base pair paired-end reads was used for sequencing on the Illumina NovaSeq platform. The raw data were filtered and de novo assembled into contigs by using CLC Genomics Workbench software (version 12.0; CLCbio). The molecular characterization was conducted using online tools, including MLST Finder 2.0 (https://cge.cbs.dtu.dk/services/MLST/) for Multilocus sequence (MLST) typing, spaTyper 1.0 (https://cge.cbs.dtu.dk/services/spatyper/) for *spa* typing, and SCCmecFinder 1.2 (https://cge.cbs.dtu.dk/services/SCCmecFinder/) for Staphylococcus cassette chromosome *mec* (SCCmec) typing. Antimicrobial resistance genes were identified using AMRFinderPlus v3.9.8. Virulence genes were mined by ABRicate v1.01 (https://github.com/tseemann/abricate) using VFDB database with 95% identity and 90% query coverage cutoffs. Additional gene homology analysis was conducted using BLAST.

Phylogenetic analysis and Bayesian evolutionary analysis

In order to investigate the evolutionary dynamics of ST22 MRSA isolates in China, genomes of additional 480 ST22 strains were downloaded from NCBI RefSeq or SRA database for comparison. A previous described method was used to infer time scaled phylogeny of ST22 strains(4). In brief, Snippy v4.6.0 was used to identify core SNPs for the ST22 genome and the BactDating R package was used to estimate node dates of ST22 strains. The recombination-corrected tree from Gubbins output and the isolation dates were used as the inputs in BactDating v1.1.

Mouse skin abscess model

BALB/c female mice aged four to six weeks old were selected for the mouse skin abscess model. *S. aureus strains* were grown for nine hours (the post-exponential phase) in fresh TSB at 37°C with shaking (220rpm), and washed twice in sterile phosphate-buffered saline (PBS) solution. Then, the mice were subcutaneously inoculated with 100μL PBS containing 1×10^8 CFU live *S. aureus* in the back skin, or injected with 100μL sterile PBS solution as the negative control (six mice in each group). The abscess length (L) and width (W) dimensions were measured by a vernier caliper daily, and the size of the abscesses was calculated using the formula: A =(L×W). Wilcoxon tests or unpaired two-tailed Student's t-tests were performed to analyze statistical significance. After measuring and recording skin abscesses for six days, all the mice were sacrificed with euthanasia. One representative mouse was selected from each group to examine the histopathological section of the skin abscess, while the other five mice were used to evaluate the bacterial burden in the skin abscess site.

G. mellonella infection model

S. aureus strains were cultured in fresh TSB for nine hours (the post-exponential phase) with shaking at 220rpm at 37°C. The cultures were then diluted with PBS to one McF bacterial suspension. *G. mellonella* (220–320 mg each) were divided into seven groups (MSSA-21, HA-MRSA ST5, HA-MRSA ST239, CA-MRSA ST59, USA300 and PBS) (n=10 *G. mellonella* in each group). *G. mellonella* were injected in the right hind paw with 10μL containing 3×10^8 CFU live S. aureus bacterial suspension. Subsequently, the *G. mellonella* were placed in a clean petri dish, and incubated in a constant temperature incubator at 37°C for three days. The number of dead *G. mellonella* was recorded every 12 hours and a survival curve was drawn. This assay was repeated in three times.

Analysis of Hemolytic activities

Lysis of erythrocytes tests were carried out as described before(5). *S. aureus* strains were cultivated for 16 h in fresh TSB at 37°C with shaking (220rpm) and centrifuged. The hemolytic activities were identified by adding 200μL supernatant samples to 800μL PBS solution containing 3% sterile rabbit red blood cells (RRBCs) and incubating at 37°C for one hour. Hemolytic capacity was determined by measuring the optical density at 600 nm using Micro ELISA Autoreader. Every sample was performed in triplicate.

Biofilm semi-quantitative assay

Biofilm semi-quantitative assays were performed as described before(5). Overnight S. aureus cultures were diluted 1:100 in TSB containing 0.5% glucose (Sigma-Aldrich, St. Louis, MO, USA), dispensed (200μL) into 96-well microtiter plates (BD Biosciences, Franklin Lakes, NJ, USA) and statically incubated at 37°C for 24 hours. The capacity of biofilm-forming was determined by measuring the optical density of stained biofilm at 600 nm with a Micro-ELISA Autoreader. The samples were tested in triplicate and this assay was repeated three times.

Quantitative Enzyme-Linked Immunosorbent Assay (ELISA) for α-Toxin

The α-toxin was detected by a *staphylococcal* α-toxin Elisa kit (Sigma-Aldrich, St. Louis, MO, United States). Overnight *S. aureus* cultures were diluted 1:200 into 4ml TSB for an additional 24 h at 37°C and adjusted to a same absorbance at OD600. Thereafter, the supernatants were collected by centrifugation for two minutes at 12,000 g and then followed the kit instructions for the ELISA. The

samples were tested in triplicate and this assay was repeated three times.

Real-time fluorescence quantitative PCR (RT-qPCR)

The expressions of the *agrA* and *RNAIII* genes in ST22 strains were evaluated by RT-qPCR with *gyrB* as an internal control. Sixteen-hour cultured S. aureus MSSA-21, HA-MRSA ST5, HA-MRSA ST239, CA-MRSA ST59, CA-MRSA USA300 and SCCmecIVa-t309 strain (MR506) were tested. Each reaction was performed in triplicate.

Statistical analysis

Unpaired two-tailed Student's t-tests and Wilcoxon tests were performed to analyze statistical significance. All data in this study were analyzed using GraphPad Prism 8.0.2 and the error bars in all graphs represented the standard deviation (mean ± SD). P values <0.05 were considered statistically significant.

1. Wang B, Xu Y, Zhao H, Wang X, Rao L, Guo Y, et al. Methicillin-resistant Staphylococcus aureus in China: a multicentre longitudinal study and whole-genome sequencing. Emerging microbes & infections. 2022 Dec;11(1):532-42. PubMed PMID: 35060838. PMCID: PMC8843102. Epub 2022/01/22. eng.
2. *CLSI*. Performance Standards for Antimicrobial Susceptibility Testing, M100, 2020.
3. *TECoAS T*. Breakpoint tables for interpretation of MICs and zone diameters, version 10.0, 2020.
4. Yu F, Cienfuegos-Gallet AV, Cunningham MH, Jin Y, Wang B, Kreiswirth BN, et al. Molecular Evolution and Adaptation of Livestock-Associated Methicillin-Resistant Staphylococcus aureus (LA-MRSA) Sequence Type 9. mSystems. 2021 Jun 29;6(3):e0049221. PubMed PMID: 34156294. PMCID: PMC8269235. Epub 2021/06/23. eng.
5. Wang X, Zhao H, Wang B, Zhou Y, Xu Y, Rao L, et al. Identification of methicillin-resistant Staphylococcus aureus ST8 isolates in China with potential high virulence. Emerging microbes & infections. 2022 Dec;11(1):507-18. PubMed PMID: 35044290. PMCID: PMC8843119. Epub 2022/01/20. eng.
